# p16/ki‐67 dual stain triage of individuals positive for HPV to detect cervical precancerous lesions

**DOI:** 10.1002/ijc.35353

**Published:** 2025-02-04

**Authors:** Diane M. Harper, Tamera Paczos, Ruediger Ridder, Warner K. Huh

**Affiliations:** ^1^ University of Michigan Ann Arbor Michigan USA; ^2^ BronxCare Health System Bronx New York USA; ^3^ Roche Diagnostics GmbH Mannheim Germany; ^4^ University of Alabama at Birmingham Birmingham Alabama USA

**Keywords:** cervical cancer screening, dual stain, HPV, triage

## Abstract

p16/Ki‐67 dual stain is a biomarker‐based test that can identify oncogenic transformation in cervical cells with higher sensitivity than cervical cytology, using the same samples taken for human papillomavirus (HPV) testing and liquid‐based cytology. Dual stain is approved by the US Food and Drug Administration (FDA) for triage of women with positive results by primary HPV testing or by HPV/cytology co‐testing and has recently been incorporated into management guidelines. In this review, we summarize the data showing the utility of dual stain in detecting precancers, reducing the number of unnecessary colposcopies, and reassuring women who test negative. We also discuss the implications of dual stain for future treatment practice and health economics.

## Graphical abstract

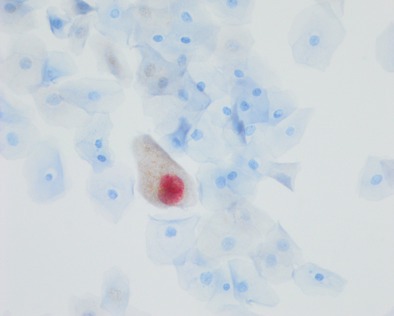

## 1 INTRODUCTION

Cervical cancer is the fourth most common cancer in women worldwide, as well as the fourth highest cause of cancer mortality.^1^ The introduction of cervical cytology to identify women and individuals with a cervix for investigation of potentially precancerous lesions led to a marked reduction in the incidence of cervical cancer in the US and elsewhere.^2^, ^3^, ^4^ However, cervical cytology is subjective and its diagnostic value is limited by substantial interobserver variability and suboptimal sensitivity.^5^, ^6^

Recognition that more than 90% of cervical cancer cases involve persistent infection with high‐risk subtypes of human papillomavirus (HPV)^7^ has led to HPV vaccination programs and changes in screening to be HPV‐based, with or without routine cytology. Women testing negative for HPV are at low risk of cervical cancer for up to 5 years.^8^, ^9^, ^10^ However, more than 90% of HPV infections are transient and do not lead to precancer. For women testing positive for HPV, a triage test that adds specificity while preserving the high sensitivity of primary HPV screening is needed.

The HPV16 and HPV18 genotypes are associated with a high incidence of lesions including cervical intraepithelial neoplasia (CIN) and adenocarcinoma in situ (AIS) that are likely to progress to invasive cancer. The incidence of CIN grade 3 or greater (CIN3+) in women with HPV16 or 18 exceeds the 4% threshold for referral to colposcopy‐directed biopsy as defined in current management guidelines in the United States of America (U.S.), while women with other high‐risk HPV genotypes need a further triage test to determine if their risk exceeds the threshold, or if the risk is low enough that they can be followed up 1 year later.^11^, ^12^

While primary HPV screening and co‐testing are more sensitive than cervical cytology in the detection of precancerous lesions, many women with transient HPV infections are still referred for unnecessary colposcopy. Where co‐testing is used and genotyping is available, women with HPV 16 and 18 genotypes are referred for immediate colposcopy; those with other genotypes are referred if their cervical cytology shows abnormal squamous cells of undetermined significance (ASC‐US) or worse using the Bethesda system.^13^ Those with normal cytology are asked to return for retesting after 12 months, and this requirement for delayed retesting causes a percentage of patients to be lost to follow‐up. Furthermore, barriers to follow‐up can disproportionately affect ethnic and racial minorities.^14^, ^15^ There is a need for a sensitive and specific test to delineate those women who can be reassured that their HPV infection is not transforming and those who need a colposcopy‐directed biopsy, as well as a need to break down barriers to receiving care, such as costs in time, effort, and patient anxiety.

In 2020, a new triage test, p16/Ki‐67 dual stain cytology (“dual stain”), was approved by the US Food and Drug Administration (FDA) to stratify certain patients with positive HPV test results by the risk of oncogenic transformation. The test was approved for use in patients screened by primary HPV or co‐testing who are positive for high‐risk HPV genotypes to delineate those who require colposcopy and those who can be reassured that they are at low risk. Clinical studies have shown that dual stain triaging can increase precancer detection rates while reducing the rate of colposcopy referral and increasing reassurance in women without precancerous lesions.^16^, ^17^, ^18^ Cost‐effectiveness analyses suggest this is achieved without increasing the overall burden on resources.^19^ Here, we summarize the evidence base supporting the clinical use of p16/Ki‐67 dual stain in the triage of women positive for HPV and its incorporation into management guidelines, particularly in the United States.

## 2 DETECTION OF ONCOGENIC TRANSFORMATION BY P16/KI‐67 DUAL STAIN

**FIGURE 1:**
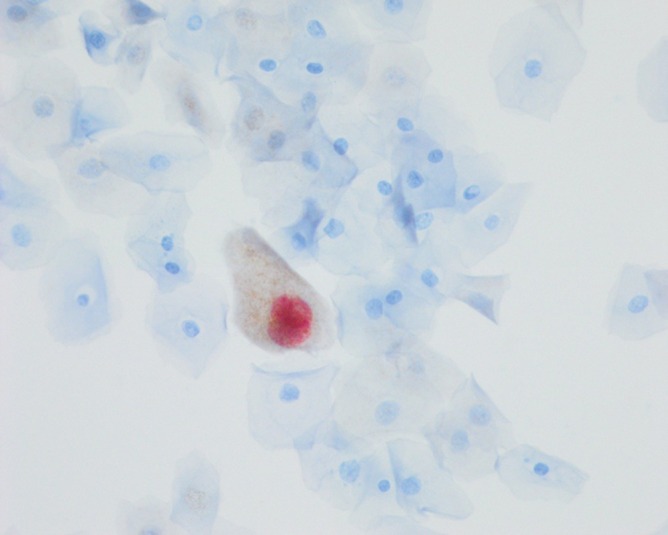
Dual stain of p16 (brown, in the cytoplasm) and Ki‐67 (red, in the nucleus) in the same cell.

p16 and Ki‐67 are proteins involved in cell‐cycle regulation and cellular proliferation. p16 is a tumor‐suppressor protein only expressed during the G0 phase of the cell cycle, while Ki‐67 is expressed in G1 through to S phase but not in G0.^20^ In normal cells, p16 and Ki‐67 are not expressed at the same time within a cell. During oncogenic transformation, the HPV oncoprotein E7 upregulates p16 to be expressed throughout the cell cycle.^21^ Detection of both p16 and Ki‐67 within the same cell therefore indicates that the oncogenic transformation process has started,^21^, ^22^ and is used clinically to confirm HPV transformation in the diagnosis of CIN on cervical biopsies.^22^, ^23^, ^24^ Simultaneous p16/Ki‐67 expression is detected by dual staining using antibodies against the two proteins: cells expressing both markers are identified by brown staining in the cytoplasm (p16) red staining in the nucleus (Ki‐67) (Figure 1).^25^ The clinically validated threshold for a positive test is a single dual‐stained, p16/Ki‐67‐positive cell on the slide.^25^, ^26^

## 3 CLINICAL APPLICATION AND VALIDATION OF P16/KI‐67 DUAL STAIN

p16/Ki‐67 dual stain is approved by the FDA in the US, as well as in other countries worldwide, for triage of women positive for HPV. Validation of dual stain for the detection of high‐grade precancers was performed by comparing it with both cervical cytology and HPV testing in a rigorous primary screening setting in 27,349 women in the prospective PALMS (Primary ASC‐US and LSIL Marker Study),^16^ where the dual stain had a higher sensitivity than cervical cytology for the detection of CIN2+ (86.7% vs. 68.5%, *p* < .001) with comparable specificity (95.2% vs. 95.4%, *p* = .15).

Subsequently, a retrospective substudy of 7727 women enrolled in the prospective ATHENA (Addressing the Need for Advanced HPV Diagnostics) trial demonstrated that dual stain triage of women testing positive for HPV would increase the number of precancers detected while reducing unnecessary colposcopies.^18^ Samples collected at enrollment were retrospectively stained by dual stain, and of the 367 CIN2+ and 248 CIN3+ cases available for retrospective testing, dual stain would have detected 258 and 182 (sensitivity of 70.3% and 74.9%), respectively, at baseline, whereas cervical cytology would only have detected 190 and 126 (sensitivity of 51.8% and 51.9%), respectively. This higher detection rate was achieved with fewer colposcopy referrals required per CIN2+/CIN3+ detected. Dual stain remained superior to cervical cytology if partial genotyping was performed, with HPV16/18 referred for colposcopy. Fewer colposcopies were required using dual stain compared with cervical cytology for each case of CIN2+ (3.81 vs. 4.73) or CIN3+ (5.40 vs. 7.13) detected. The addition of partial genotyping increased the number of CIN2+ and CIN3+ cases detected (sensitivity of 82.8% and 86.8%, respectively) but also resulted in more colposcopy referrals per CIN2+/CIN3+ detected.^18^ Dual stains showed superior performance to cervical cytology as triage of women positive for HPV, with or without the addition of partial genotyping.

**FIGURE 2:**
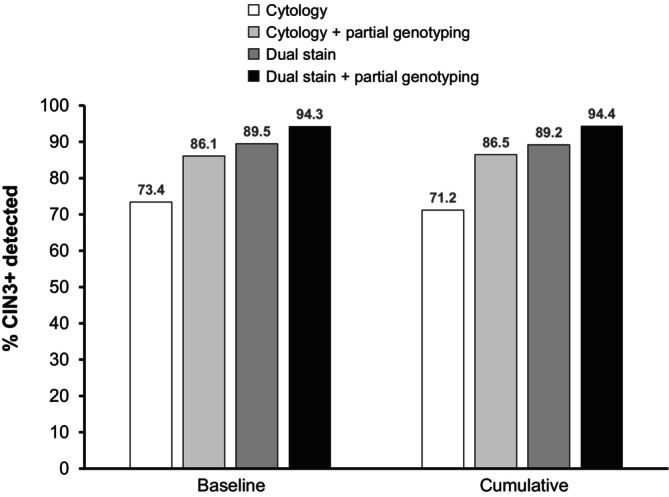
Sensitivity of different triage methods for detection of CIN3+ at baseline or cumulative over 1 year in the IMPACT trial.^28^ CIN, cervical intraepithelial neoplasia.

The IMproved Primary screening And Colposcopy Triage (IMPACT) trial demonstrated the benefit of dual‐stain triage following HPV testing or co‐testing in a routine cervical cancer screening population in the US. A total of 35,263 women were screened by HPV testing and cervical cytology, with referral for colposcopy for those with positive HPV and/or abnormal cytology results.^27^, ^28^ Of the 5250 women positive for HPV, 4927 also had valid dual stain results; dual stain would have detected 205/229 (89.5%) cases of CIN3+ compared with 168/229 (73.4%) for cervical cytology.^28^ An approach using colposcopy for HPV16/18 and dual‐stain triage for other high‐risk genotypes would have identified 216/229 CIN3+ cases (94.3%) compared with 198/229 (86.5%) for genotyping plus cervical cytology (Figure 2).

In a prospective observational study of 3225 women positive for HPV with partial genotyping at Kaiser Permanente Northern California (KPNC), 12.0% of those with positive dual stain were found to have CIN3+ compared with 10.3% of those with abnormal cervical cytology (*p* = .05).^29^ In the STRIDES (STudying Risk to Improve DisparitiES in Cervical Cancer in Mississippi) study, there were 88 cases of CIN3+ over 1 year in 768 women with positive dual stain (risk: 11.5%) and only eight cases in 1154 with negative dual stain (risk: 0.7%) (data from guidelines publication).^30^

Data from the IMPACT trial indicate that triage of women positive for HPV by dual stain would have resulted in significantly fewer women being referred for colposcopy compared with HPV genotyping and cervical cytology (49% vs. 56%, *p* < .001), leading to significantly higher colposcopy efficiency (4.09 vs. 5.35 colposcopies per CIN2+ detected, *p* < .001).^28^ The negative predictive value of dual stain for detection of CIN3+ within 1 year in women positive for HPV was 98.6%, indicating a high degree of certainty associated with a negative result.^28^ Together with the data from ATHENA and other studies, this shows that triage of women positive for HPV by dual stain can efficiently detect oncogenic transformation while reassuring women who test negative that their risk of cervical cancer is low.

Numerous studies across various geographic regions have consistently demonstrated high sensitivity and specificity of dual stain when used for the triage of HPV‐positive women^28^, ^29^, ^30^, ^31^, ^32^, ^33^, ^34^, ^35^, ^36^, ^37^, ^38^, ^39^, ^40^, ^41^, ^42^, ^43^, ^44^; performance characteristics for the detection of CIN2+ and CIN3+ are summarized in Table 1.

**TABLE 1:** Performance of p16/Ki‐67 dual stain cytology for triage of HPV positive women.

		CIN2+		CIN3+	
Study reference	HPV‐positive women (*n*)	Sensitivity (%)	Specificity (%)	Sensitivity (%)	Specificity (%)
Luttmer et al., 2016^31^	535	85.5	60.0	93.8	51.2
Yu et al., 2016^32^	463	92.7	52.7	95.0	47.7
Ebisch et al., 2017^33^	247	86.3	73.1	92.1	61.4
Stanczuk et al., 2017^34^	340	85.0	76.7	N/A	N/A
Wright et al., 2017^18^	3338	70.3	75.6	74.9	74.1
Wentzensen et al., 2019^29^	3225	82.8	55.7	88.6	53.1
Rossi et al., 2021^35^	3147	75.2	74.8	80.6	N/A
Wright et al., 2021^28^	5250	86.5	57.5	89.5	54.0
Chen et al., 2022^36^	10,500	82.8	51.6	89.5	47.2
Gustinucci et al., 2022^37^	4202	75.8	80.2	85.0	N/A
Ovestad et al., 2023^38^	1415	82.7	65.9	84.4	63.5
Trzeszcz et al., 2023^39^	1086	94.1	52.1	97.1	45.1
Clarke et al., 2024 (IRIS)^30^	3617	N/A	N/A	87.3	54.6
Clarke et al., 2024 (STRIDES)^30^	1922	N/A	N/A	91.7	62.8

*Note*: Numbers for IRIS and STRIDES studies provided in the supplemental material of Clarke et al., 2024.^30^

Abbreviations: CIN, cervical intraepithelial neoplasia; CIN2 (3)+, CIN2 (3) or worse; N/A, not available.

## 4 LONGITUDINAL RISK STRATIFICATION

The risk of CIN2/3 developing over several years is an important parameter in considering the performance of a triage test. The American Society for Colposcopy and Cervical Pathology (ASCCP) risk‐management guidelines use immediate risk of CIN3+ for consideration of clinical action such as colposcopy or treatment and 3/5‐year risk of CIN3+ for longer term surveillance recommendations.^12^

Wentzensen et al. (2019) evaluated the risk of CIN3+ in 3225 women positive for HPV at KPNC with cervical cytology who also had a valid dual stain result.^29^ Dual stain showed a superior risk stratification than cervical cytology, with or without HPV genotyping. Women with positive dual stains showed a 12.0% risk of CIN3+ within one screening cycle, compared with 10.3% for cervical cytology (*p* = .005). The negative predictive value of dual stain for the detection of CIN3+ was 98.5%.^29^

**FIGURE 3:**
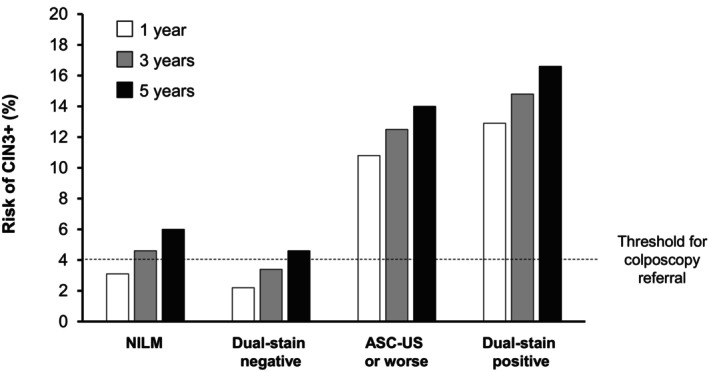
1‐, 3‐, and 5‐year risk of CIN3+ by cervical cytology or dual stain results at baseline in a KPNC study.^45^ ASC‐US, atypical squamous cells of undetermined significance; CIN, cervical intraepithelial neoplasia; NILM, negative for intraepithelial lesion or malignancy.

**FIGURE 4:**
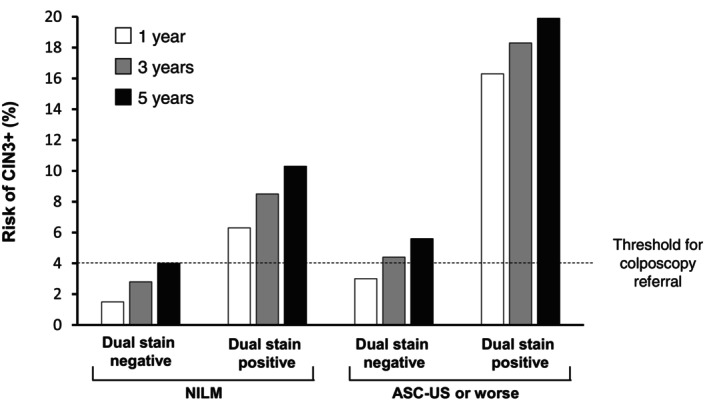
1‐, 3‐, and 5‐year risk of CIN3+ by cervical cytology combined with dual stain results.^45^ ASC‐US, atypical squamous cells of undetermined significance; CIN, cervical intraepithelial neoplasia; NILM, negative for intraepithelial lesion or malignancy.

In a prospective study of 1549 women positive for HPV, also at KPNC, positivity by dual stain was associated with a significantly higher cumulative risk of CIN3+ compared with abnormal cervical cytology (Figure 3).^45^ Over 5 years, women testing positive for HPV with normal cervical cytology (NILM) who were positive by dual stain had a consistently higher risk of CIN3+ than those with ASC‐US who were negative by dual stain (Figure 4). In women negative by dual stain, the risk remained below that of those with NILM throughout the 5 years, and below the 4% threshold for colposcopy referral beyond year 3.^45^ The authors suggested that women with negative dual stain results may be reassured for at least 3 years. However, additional longitudinal studies will be needed to further prove the long‐term negative prediction of negative dual stain results in HPV‐positive women before expanding the retesting time interval beyond the currently recommended 1 year period.

## 5 INCORPORATION OF P16/KI‐67 DUAL STAIN INTO MANAGEMENT GUIDELINES

While regular screening is effective in reducing disease and mortality from cervical cancer, there is recognition that more effective strategies are needed to ensure that resources and treatment reach those at highest risk of precancer and that women at low risk are not asked to go for unnecessary additional testing. In the US and other countries, such as the UK^46^ and Germany,^47^ the effectiveness of dual stain in detecting oncogenic transformation is being increasingly recognized. The Enduring Consensus Cervical Cancer Screening and Management Guidelines Committee recently released guidelines on dual stain,^30^ based on findings from two cohorts of patients, one from KPNC^29^ that included the dual stain implementation study and a subset of the IRIS (Improving Risk Informed HPV Screening) cohort,^48^ and one from the STRIDES study.^15^ Both the IRIS and STRIDES data on dual stain are published in the guidelines publication.^30^ The guidelines recommended dual stain for triage of women positive for HPV in the different settings shown below.

### 5.1 2024 Enduring Guidelines Update: Triage of women positive for HPV without genotyping

In the absence of HPV genotyping, dual‐stain triage is acceptable, with colposcopy recommended for those testing positive by dual stain, and 1‐year return recommended for those testing negative by dual stain.^30^ Based on KPNC data, triage with dual stain would result in 12% fewer colposcopies and 40% fewer years to diagnose CIN3+ than triage with cervical cytology.

### 5.2 2024 Enduring Guidelines Update: Triage of women positive for HPV when limited genotyping is provided by the screening test

With limited HPV genotyping, dual stain is similarly acceptable, with colposcopy recommended for all individuals testing positive for HPV 16/18. For those testing positive for 12 other high‐risk HPV genotypes, colposcopy is recommended for those testing positive by dual stain and 1‐year return for those testing negative by dual stain. Although women who test positive for HPV16/18 but are negative by dual stain remain below the risk threshold for CIN3+, all those positive for HPV16/18 are recommended to be referred for colposcopy until further data are available, regardless of the dual stain result.^30^ Based on KPNC data, in modeling exercises using 100,000 women, the cumulative number of years before CIN3+ is diagnosed is less with dual stain than with cytology when reflexing after a positive high‐risk HPV test (105 vs. 136 years).

### 5.3 2024 Enduring Guidelines Update: Triage of women positive for HPV in a co‐testing setting

In a co‐testing setting, the dual stain is acceptable for triage of individuals with HPV‐positive test results and NILM, ASC‐US, and low‐grade squamous intraepithelial lesion (LSIL) by cervical cytology. In the absence of genotyping, colposcopy is recommended for those with NILM, ASC‐US, or LSIL cytology who test positive by dual stain and 1‐year return for those with NILM, ASC‐US, or LSIL cytology who test negative by dual stain. If limited genotyping is available, colposcopy is recommended for all patients testing positive for HPV 16/18.^30^ The immediate CIN3+ risks of those with NILM, ASC‐US, or LSIL cytology who tested positive for dual stain were 4.1%–6.6%. The immediate CIN3+ risks of HPV‐positive women with NILM, ASC‐US, or LSIL cytology who tested negative by dual stain were 0.53%–0.87% and the 3‐year risks were 0.92%–1.6%. Similar data were seen in the STRIDES cohort, although follow‐up is not yet complete. Based on KPNC data, dual stain triage in a cotesting setting results in 11% fewer total colposcopies and 64% fewer years of CIN3+ diagnoses compared with cotesting alone.

Dual stain is not recommended in individuals with cytology results of atypical squamous cells—cannot rule out high‐grade (ASC‐H), atypical glandular cells—cannot rule out high‐grade (AGC), or high‐grade squamous intraepithelial lesion (HSIL), and if obtained, should not guide management.

### 5.4 2024 Enduring Guidelines Update: Follow‐up after abnormal results, colposcopy, or treatment (surveillance settings)

When women who tested positive for HPV are being followed after abnormal screening test results that did not require colposcopy or treatment, it is acceptable to use dual stain according to the guidelines for management of an initial abnormal screening test result.^30^

Although dual stain does not currently feature in European guidelines,^49^ its superiority over cervical cytology in the triage of women positive for HPV is being increasingly recognized.^46^, ^47^ Additionally, the WHO guidelines for cervical cancer screening now include dual stain testing to triage women after a positive HPV test result, enhancing early detection with a more sensitive and specific method for identifying high‐risk cases globally.^50^

## 6 IMPLEMENTATION IN CLINICAL PRACTICE AND LIMITATIONS

Implementing dual stain testing in cervical cancer screening, ordered by physicians as a reflex test after a positive primary HPV test, enhances early detection of CIN2+/CIN3+ lesions and reduces unnecessary follow‐ups and treatments, either in combination with partial HPV 16/18 genotyping results or without. Furthermore, future studies will show how the potential combination of dual stain with extended HPV genotyping may further optimize cervical cancer screening and patient management algorithms.

As with every new technology, appropriate training is important to achieve accurate and reliable results. Several studies have been performed to analyze the reproducibility of dual stain cytology in clinical cytology laboratories. While some studies demonstrated good to excellent reproducibility after initial training, with almost identical clinical performance of novice evaluators compared with reference evaluations,^26^, ^51^ other studies highlighted the need for more detailed microscope training sessions with troubleshooting slide review of discordant cases to ensure high inter‐reader reproducibility and accuracy.^52^, ^53^ Weak intensity of p16 staining, occasional background staining, and low numbers of dual‐stained cells (i.e., 1 or 2 cells per slide) were reported as characteristics of difficult cases, which may lead to lower initial reproducibility between readers.

Another limitation of dual stain is that its use on self‐collected clinical specimens, similar to cervical cytology, most likely will not represent a promising approach. Although a very limited number of studies have shown that it may be feasible in principle, the likelihood that the clinical sensitivity of dual stain on self‐collected vaginal samples is sufficient for the initial triage of HPV‐positive women is low.^54^, ^55^

## 7 HEALTH ECONOMICS

The economic impact of cervical cancer is high,^56^ and early, more efficient detection can be expected to reduce healthcare and other costs associated with the disease. Using a payer‐perspective Markov microsimulation model, the impact of dual stain following co‐testing on healthcare costs in the US has been found to be cost‐effective.^19^ Based on test performance data from the IMPACT trial, the incremental cost‐effectiveness ratio (ICER) of dual stain used as a reflex test after primary high‐risk HPV genotyping and cervical cytology co‐testing were $19,892 (95% CI $10,426–$61,987) per quality‐adjusted life year and $21,891 (95% CI $10,737–$85,502) per life‐year gained; $50,000 to $150,000 are considered cost‐effective. With the use of dual stain reflex testing after co‐testing, costs associated with screening and medical costs increased, while costs associated with invasive cervical cancer decreased.^19^

Studies in several other countries have demonstrated the economic utility of p16/Ki‐67 dual stain. A German study considering budget impact over 10 years^57^ and a Belgian study over 6 years^58^ both concluded that dual stain was associated with lower annual costs compared with cervical cytology. The Belgian study estimated that replacing cervical cytology with dual stain as triage for women positive for HPV would reduce the healthcare costs associated with screening by 22% for women aged 30–65 and by 21% for women aged 25–65.^58^ A further study estimated that the replacement of cervical cytology triage with dual stain could lead to a saving of €15 million to the Belgian healthcare system based on the use of 5‐year screening intervals. Two studies in Thailand concluded that HPV screening with dual stain triage offered the most cost‐effective option.^59^, ^60^

## 8 CONCLUSIONS

Numerous studies worldwide have demonstrated the superior performance of dual stain compared with cervical cytology for the triage of women positive for HPV. Dual stain can result in fewer unnecessary colposcopy referrals, earlier detection of precancerous lesions, and reduced likelihood of patients being lost to follow‐up before their cancer is detected. For women positive for HPV who test negative by dual stain, the negative predictive value for detection of CIN3+ of 98%–99%^29^ provides substantial reassurance.

Management guidelines for cervical cancer are evolving, and dual stain promises to be central to future management. ASCCP guidelines for the use of dual stain in the triage of individuals positive for HPV have been released,^30^ and it can be expected that these guidelines will be adopted in other parts of the world. Health economic data indicate dual stain to be among the most cost‐effective methods of triage.^19^, ^57^, ^58^, ^59^, ^60^

Because dual stain utilizes the same samples as physician‐collected HPV testing and cervical cytology, it can be adopted by clinical laboratories with minimal disruption. Further development of dual stain technology may enable artificial intelligence (AI)‐assisted automation, improving workflow efficiency and interpretation of samples. In one study, cloud‐based AI reading of dual stain was shown to provide equivalent sensitivity with higher specificity than manual reading.^61^

Overall, dual stain represents an effective method for triage of women positive for HPV, using the same samples and with the ability to detect more precancerous lesions than liquid‐based cytology while at the same time reducing the number of unnecessary colposcopies and, importantly, providing greater reassurance for women testing negative, particularly combined with partial genotyping. Several studies indicate that dual stain has the potential for cost savings in healthcare systems compared with cervical cytology when used as triage after a positive HPV test. Dual stain is approved in the US for triage of women positive for HPV and has recently been incorporated into US guidelines, with other countries and regions expected to follow.

## AUTHOR CONTRIBUTIONS

**Diane M. Harper:** Conceptualization; funding acquisition; writing – original draft; writing – review and editing. **Tamera Paczos:** Writing – review and editing. **Ruediger Ridder:** Conceptualization; funding acquisition; writing – original draft; writing – review and editing; methodology. **Warner K. Huh:** Conceptualization; writing – review and editing.

## CONFLICT OF INTEREST STATEMENT

T. P. has been a speaker and consultant to Roche Diagnostics. R. R. is an employee of Roche Diagnostics and reports minority stock ownership in Roche Holding AG. The other authors have no conflicts to declare.
